# The role of vagus nerve stimulation in modulating Parkinson’s disease via the microbiota-gut-brain axis: a comprehensive review

**DOI:** 10.3389/fneur.2025.1643305

**Published:** 2025-09-22

**Authors:** Lingqing Yang, Shiyu Fan, Li Sun, Jingru Han, Meng Wang, Tao Yu

**Affiliations:** ^1^The First Affiliated Hospital of Tianjin University of Traditional Chinese Medicine/National Clinical Medical Research Center of Acupuncture, Tianjin, China; ^2^Tianjin University of Traditional Chinese Medicine, Tianjin, China; ^3^Tianjin Xiqing District Hospital of Traditional Chinese Medicine, Tianjin, China

**Keywords:** vagus nerve stimulation, Parkinson’s disease, microbiota-gut-brain axis, mechanism, intestinal flora

## Abstract

As the global aging trend intensifies, the incidence of neurodegenerative diseases, including Parkinson’s disease (PD), is increasing year by year. Currently, there is no effective cure for PD. Therefore, exploring safe and effective therapeutic targets is of utmost importance. Previous studies have shown that modulation of vagus nerve (VN) activity, a key communication pathway between the brain and the gut, may produce therapeutic effects in PD and influence its disease course by regulating the gut microbiota, brain plasticity, neuroimmune, and neuroendocrine systems, while the nerve itself also plays a complex role that can contribute to pathological processes like disease propagation. This review comprehensively summarizes the potential mechanisms by which vagus nerve stimulation (VNS) intervenes in PD may influence the microbiota-gut-brain axis (MGBA), including the regulation of gut microbiota composition and metabolites, inhibition of central and peripheral neuroinflammatory responses, modulation of hypothalamic–pituitary–adrenal (HPA) axis function, enhancement of brain region functional connectivity and neurotrophic factor secretion, and explores its potential value in translating into clinical therapeutic strategies. This study is the first to integrate the MGBA theory with VNS technology, revealing its cross-system regulatory network in intervening PD and providing new ideas for breaking through the limitations of traditional treatments.

## 1 Introduction

Parkinson’s disease (PD) is a common neurodegenerative disorder that primarily affects the middle-aged and elderly population. It is characterized by the accumulation of α-synuclein (α-Syn) in the central and peripheral autonomic nervous systems, which contributes to the gradual degeneration of dopaminergic (DA) neurons in the substantia nigra pars compacta (SNc) of the midbrain (1). The clinical manifestations of PD are diverse, encompassing a range of motor symptoms, such as bradykinesia, resting tremor, rigidity, and postural instability. In addition, non-motor symptoms, including psychiatric symptoms like anxiety and depression, as well as gastrointestinal symptoms, are also frequently observed in patients with PD (2). In recent years, the incidence of PD has been on a continuous rise. The global prevalence of PD in individuals under 50 years of age is 5.50%, while it significantly increases to 80% in those aged 80 years and above (3). This undoubtedly places a heavy burden on patients’ families and society. Currently, the treatment of PD mainly focuses on symptom control and slowing disease progression. Traditional therapies include levodopa medication and neurosurgical interventions. However, the therapeutic efficacy of levodopa frequently diminishes over time due to disease progression and pharmacokinetic changes, alongside worsening side effects. While neurosurgical interventions (e.g., lesioning, Deep Brain Stimulation) can alleviate symptoms, they involve irreversible neuronal damage (4). Therefore, the search for a safe and effective alternative therapy is imperative.

In recent years, the microbiota-gut-brain axis (MGBA) has emerged as a crucial research domain in the treatment of PD. The MGBA tightly connects the gut with the brain, forming an interactive network of bidirectional communication between the gastrointestinal system (GIS) and the central nervous system (CNS) (5). The gut microbiota, a key component of the MGBA, is closely associated with gastrointestinal dysfunction, CNS inflammation, and DA degeneration (6). The gut microbiota exerts significant regulatory effects on the brain-gut axis through immune, neuroendocrine, and direct neural mechanisms, influencing neurodevelopment, brain function, and behavior (7, 8). Research has shown that enteric nervous system (ENS) degeneration is closely linked to PD (9), with the loss of enteric neurons being implicated in the development of PD-related dysfunction. Changes in the function, connectivity, mitochondria, and/or α-Syn of enteric neurons, as well as alterations in their extrinsic innervation, may underlie the gastrointestinal dysfunction observed in PD patients (10). Sampson et al. comprehensively elucidated the critical role of the MGBA in the progression of PD, demonstrating that PD-associated alterations in the gut microbiota can lead to motor deficits, promote microglial activation, and facilitate α-Syn aggregation (11). Moreover, short-chain fatty acids (SCFAs), metabolites of the gut microbiota, exacerbate neuroinflammation and motor impairments in PD models, suggesting that SCFAs may act as potential molecular mediators in gut-brain signaling. In summary, the MGBA influences the pathogenesis and progression of PD through neuroimmune and neuroendocrine pathways.

The vagus nerve (VN), as the tenth cranial nerve, has been established as a crucial mediator of bidirectional communication between the brain and the gut (12). In recent years, the VN has been shown to be closely associated with the pathogenesis and progression of PD (13). A systematic review by Abdelnaby revealed that PD patients exhibit a degree of VN atrophy and confirmed the diagnostic value of neurosonography in PD (14). An increasing number of studies in recent years have supported Braak’s hypothesis that the VN serves as a “highway” for the propagation of pathological α-Syn from the gut to the brain, thereby elucidating the pathogenesis of PD (1, 15). Consequently, vagotomy can prevent the prion-like transmission of α-Syn, thereby reducing the risk of developing PD (16). Mondal et al. demonstrated that vagus nerve stimulation (VNS) effectively improves two-dimensional spatiotemporal gait parameters in PD patients, suggesting that VNS holds therapeutic potential for PD (17).

**Figure 1:**
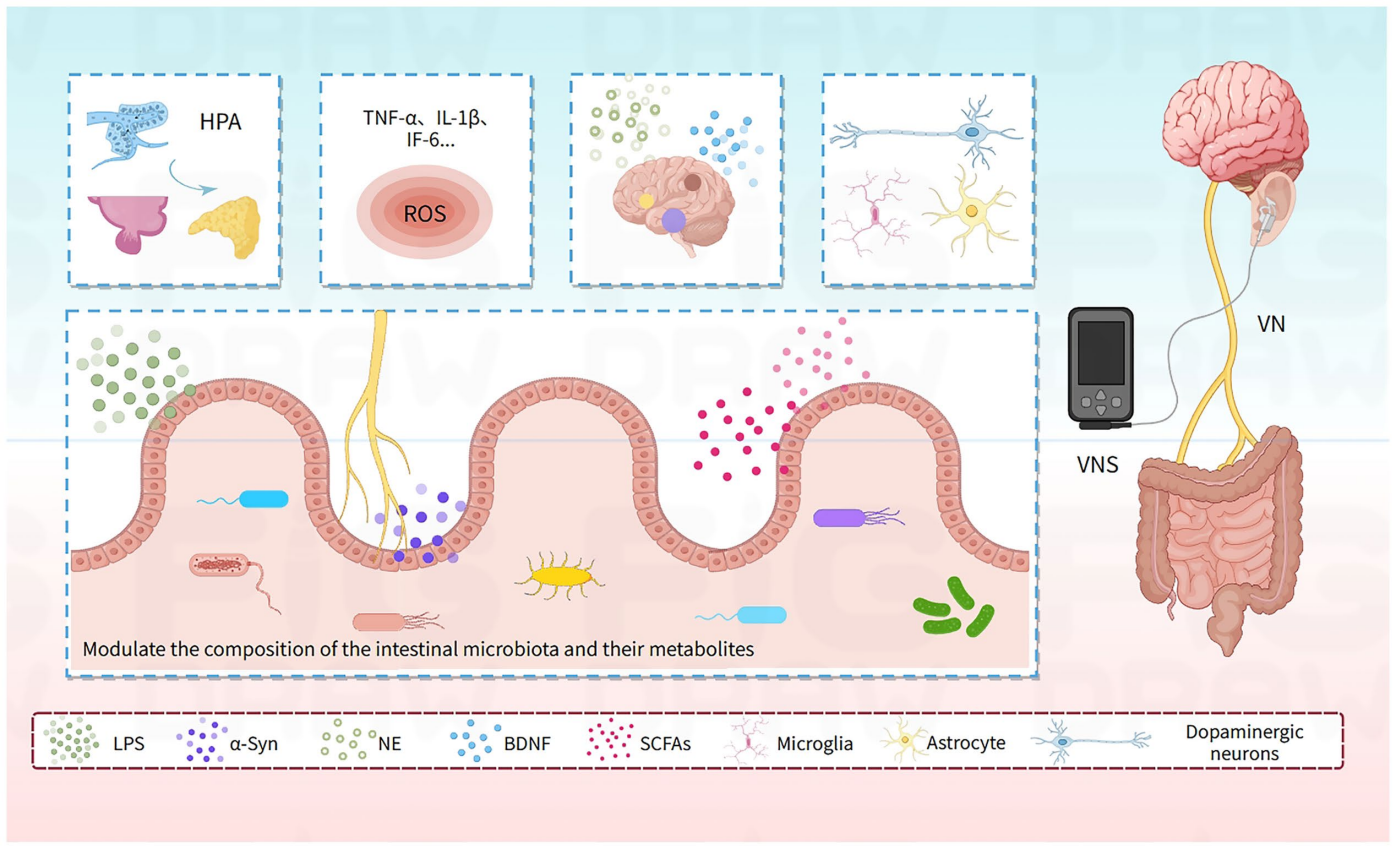
Multidimensional mechanisms of vagus nerve stimulation in Parkinson’s disease via the microbiota-gut-brain axis. α-Syn, α-synuclein; LPS, lipopolysaccharide; SCFAs, short-chain fatty acids; TNF-α, tumor necrosis factor-alpha; IL-1β, interleukin-1 beta; ROS, reactive oxygen species; BDNF, brain-derived neurotrophic factor; HPA, (hypothalamic–pituitary–adrenal) axis; VN, vagus nerve.

In summary, the VN, as a critical relay station of the MGBA, plays a significant role in the pathogenesis and progression of PD. However, the specific molecular mechanisms underlying the intervention of PD by VNS via the MGBA remain unclear, despite its emerging status as a novel therapeutic modality. Therefore, this study aims to integrate preclinical research with translational medical evidence to provide a mechanism-driven theoretical basis for the transition of VNS technology from experimental exploration to personalized precision therapy (Figure 1).

VNS modulates PD pathology through two synergistic pathways: (1) Peripheral (Gut) Effects: Reshapes gut microbiota composition and metabolites (↓ LPS, ↑ SCFAs), reduces intestinal inflammation and α-Syn expression, and inhibits α-Syn propagation to the CNS via the VN. (2) Central (Brain) Effects: Attenuates neuroinflammation (↓ microglia/astrocyte activation; ↓ TNF-α, IL-1β, ROS), enhances brain connectivity, ↑ BDNF secretion, and protects dopaminergic neurons. Simultaneously, VNS normalizes HPA axis function (↓ cortisol), ameliorating gut barrier dysfunction and synergistically amplifying therapeutic outcomes.

## 2 The relationship between the PD and the gut-microbiome-brain axis

### 2.1 Relationship between PD and brainstem nuclei

As a key structural component of the MGBA, the VN contains circuitry involving critical brainstem nuclei: the Nucleus Tractus Solitarius (NTS), Locus Coeruleus (LC), and dorsal motor nucleus of the vagus (DMV) (18, 19). The NTS is a vital polysynaptic hub in the human brainstem, located ventrolaterally to the dorsal motor nucleus of the VN. It serves as a major integration site for central sensory afferent neurons and is closely associated with various neural and endocrine systems (20). The NTS also plays a significant role in the pathogenesis of PD. Previous studies have suggested that PD-related respiratory failure may be associated with neuronal degeneration in the NTS. Their research identified impaired substantia nigra pars compacta (SNpc)—periaqueductal gray (PAG) - caudal nucleus of the solitary tract (cNTS) pathways in PD models, elucidating the loss of Phox2b-expressing neurons or hypoxia-activated neurons in the cNTS and subsequent respiratory dysfunction during hypoxic stimulation (21). Sun et al. demonstrated that the administration of 3,4-dihydroxyphenylacetaldehyde (DOPAL) to the VN in rats resulted in the transport of DOPAL and/or higher molecular weight α-Syn mediated by DOPAL via vagal afferent nerves to the nodose ganglion (NG) and NTS (22). This caused significant ultrastructural changes in the NG and NTS and further exacerbated PD-like autonomic dysfunction in rats.

The LC is the primary site in the brain for the production of norepinephrine (NE). NE-producing neurons in the LC widely project to many regions of the CNS to modulate functions such as attention, arousal, pain, mood, and stress responses (23, 24). Neuronal degeneration in the LC and NE signaling dysfunction are common in PD patients. The LC is also one of the earliest brain regions affected by α-Syn pathology, although the mechanisms by which α-Syn affects these neurons remain unclear (25). According to Braak’s staging of α-Syn pathology, the LC is affected early in the disease course (stage 2), before the deposition in the DA substantia nigra (SN) (stage 3), and significant loss of LC-NE neurons becomes apparent as the disease progresses (26). NE neurotransmission dysfunction is associated with non-motor symptoms of PD, including sleep disturbances, anxiety, depression, and cognitive decline. Importantly, central NE deficiency may contribute to chronic inflammation and disease progression in PD (27). A study by Butkovich et al. revealed the mechanisms by which increased α-Syn expression affects central NE transmission and related behaviors (28). The results showed that overexpression of α-Syn and the formation of oligomers have cytotoxic effects, upregulate local inflammatory markers, induce LC fiber degeneration, disrupt striatal dopamine metabolism, and exacerbate non-motor behaviors in PD. Additionally, comprehensive proteomic analysis of LC tissue from PD patients has proposed that important molecular pathogenic pathways in PD include mitochondrial dysfunction, oxidative stress, and cytoskeletal dysregulation. This study also highlighted the potential pathogenic roles of certain proteins in the LC (e.g., serine/threonine-protein kinase PAK3, mitochondrial ribosomal protein MRPS6, calcium-modulating protein regucalcin, and microtubule-associated protein KTN1) and confirmed that aminoacyl-tRNA biosynthesis pathways may also be involved in the pathogenesis of PD (29).

### 2.2 Dysregulation of the HPA axis aggravates the progression of PD

The hypothalamic–pituitary–adrenal (HPA) axis is a critical neuroendocrine pathway within the MGBA. Dysfunction of the HPA axis is primarily characterized by alterations in peripheral cortisol levels and/or abnormal cortisol responses to stressful life events. Excess glucocorticoids (GCs) can induce neuronal damage in brain regions such as the hippocampus and SN through several mechanisms, including mitochondrial dysfunction, inhibition of inhibitory and excitatory synaptic transmission, and disruption of microglial or astrocytic functions, thereby exacerbating the progression of PD (30–33). Moreover, overactivation of the HPA axis can trigger oxidative stress and modulate the diversity of gut microbiota via GCs, leading to impaired intestinal barrier function and gastrointestinal dysfunction (34). Patients with PD often exhibit GC dysregulation, which can result in abnormal gut microbial metabolism, disruption of intestinal mucosal morphology, and mediation of intestinal inflammatory responses (35). These changes can subsequently affect brain function. Research has shown that GC dysregulation can lead to persistent neuroinflammation and oxidative stress in the brain, causing damage to DA neurons and worsening PD symptoms (36). However, some researchers have proposed that GCs have a bidirectional regulatory role in PD. Specifically, GCs can induce Parkin expression via the cAMP-response element-binding protein (CREB) pathway, exerting neuroprotective effects and preventing the death of DA neurons (37). Thus, the gut microbiota and neuroendocrine system interact with each other, and their combined effects influence both motor and non-motor symptoms in PD patients.

### 2.3 The close relationship between intestinal microbiota and PD

In the 1980s, the first reports of Lewy body pathology in the ENS provided evidence for ENS and gut microbiota dysfunction in PD (38). Some researchers have proposed that the pathological mechanisms of PD may originate in the gut, with α-Syn pathology spreading to the brain in a bottom-up manner (39). Shannon et al. found that α-Syn had already accumulated extensively in enteric neurons in PD patients before the onset of motor symptoms through colonic biopsies (40). Additionally, increased intestinal permeability has been observed in the early stages of PD, which may be related to the increased accumulation of α-Syn in the gut, triggering intestinal inflammation (41). Statistics show that approximately 60–80% of PD patients suffer from ENS dysfunction, including chronic constipation, drooling, and dysphagia, with significantly higher prevalence than in non-PD populations (42, 43). These non-motor symptoms often precede motor symptoms (44). In recent years, Gastrointestinal Dysfunction Scale for PD (GIDS-PD) is widely used in clinical evaluation of PD. Camacho et al. showed that intestinal dysfunction may be a phenotypic feature of PD subgroups, which has implications for patient stratification and management (45). Bissacco et al. stated that the GIDS-PD score was directly related to non-motor dysfunction in PD patients (46). Nowak et al. confirmed the reliability of constipation and irritable bowel subindicators in the assessment of PD (47). Moreover, ENS dysfunction may have already accelerated PD pathology up to 10 years before the onset of clinical symptoms (48). In recent years, the role of gut microbiota and their metabolites in the progression of PD has attracted widespread attention from the scientific community. There is now substantial evidence that as PD progresses, the number of gut microbes changes, and alterations in microbial community structure are associated with disease severity (49). Huang et al. found that gut microbiota dysregulation can occur in the prodromal stage of PD, such as a reduction in short-chain fatty acid (SCFA)-producing bacteria (*Lachnospira* and *Butyricicoccus*) and butyrate-producing *[Eubacterium]_ventriosum_*group (50). This condition is more severe in PD patients with rapid eye movement sleep behavior disorder (RBD). This may compromise the integrity of the intestinal barrier, leading to increased intestinal permeability, activation of intestinal immune responses, and accumulation of α-Syn in the gut, ultimately triggering neuroinflammation and pathological processes in PD (51). A cross-sectional study showed that PD patients had higher abundance of three phyla (*Proteobacteria, Verrucomicrobia,* and *Actinobacteria*) and five genera (*Akkermansia*, *Enterococcus*, *Hungatella*, and two *Ruminococcaceae*) in their gut microbiome. In patients with longer disease duration, the abundance of *Fournierella* and *DTU089 (Ruminococcaceae)* increased, while that of *Roseburia (Lachnospiraceae)* decreased. Additionally, the composition of gut microbiota also varied among patients with different motor subtypes (52).

In addition, metabolites of the gut microbiota, such as SCFAs and lipopolysaccharides (LPS), have been shown to play important roles in the pathogenesis and progression of PD. A healthy gut microbiota often promotes the integrity of the blood–brain barrier (BBB) by regulating tight junction protein expression mediated by SCFAs (e.g., occludin and claudin-5) (53). SCFAs also play a crucial role in maintaining the integrity of the intestinal barrier by preventing microbial translocation, thereby alleviating local intestinal inflammation, systemic inflammation, and neuroinflammation (54). LPS, an endotoxin produced by Gram-negative bacteria, interacts with immune cells in the bloodstream, upregulating the expression of pro-inflammatory cytokines (e.g., tumor necrosis factor-α (TNF-α) and interleukins) and systemic inflammation (55). In individuals with PD, certain microbial compositions (e.g., dysbiosis) have been found to stimulate the production of inflammatory cytokines and LPS, leading to intestinal epithelial damage and compromised barrier integrity (56). Studies have also shown that LPS can induce a structurally unique self-replicating α-Syn fibril strain in mice, which triggers a signature pattern of synucleinopathy similar to that induced by wild-type α-Syn commonly observed in PD (57). In addition, gut microbiota metabolites derived from dietary polyphenols (e.g., phenolic sulfates) can cross the BBB and directly modulate microglia activity and neuroinflammation (58, 59). For instance, pyrosulfate (Pyr-sulf) enhances BBB integrity while suppressing nuclear factor-κB (NF-κB) signaling pathways (60, 61). Clinical studies have further confirmed these metabolites’ presence in cerebrospinal fluid (62). Therefore, maintaining gut homeostasis, ensuring normal ENS function, and regulating the quantity and community structure of gut microbiota are of great importance for the prevention and treatment of PD.

### 2.4 Neuroinflammation in PD

PD is characterized by the slow and progressive degeneration of DA neurons in the SNc. Neuroinflammatory mechanisms are likely to be one of the causes of neuronal degeneration. Multiple studies have demonstrated that alterations in the peripheral immune system precede the emergence of classic motor symptoms in PD patients. PET imaging reveals microglia activation in the SN before motor symptoms appear, while peripheral immune cells (e.g., CD8 + T cells and monocytes) can infiltrate the central nervous system through compromised BBB (63). The pathological process of PD may originate from inflammation in the peripheral nervous system surrounding visceral organs and progress to the brainstem and SNc via the VN (64). Postmortem analyses of PD patients have shown significant increases in activated microglia, HLA-positive microglia, and astrocyte density in the SNc (65–67). These proliferating and activated glial cells induce inflammatory mediators, which subsequently cause oxidative damage and accelerate the degeneration of DA neurons in the SNc. It has been reported that pro-inflammatory changes are present in the blood and cerebrospinal fluid (CSF) of PD patients. Levels of TNF-α, interleukin 1β (IL-1β), and interleukin 6 (IL-6) are elevated in the CSF samples of PD patients (68, 69). Antibodies that recognize various components of DA neurons, including the products of dopamine oxidation, have been identified in the serum of PD patients (70). Studies have also shown that the number of CD4 + and CD45RA + T cells (representing naive lymphocytes) is reduced in the serum of PD patients, while the number of CD4 + and CD45RO + T cells (representing activated T cells) is increased, indicating that peripheral lymphocytes are activated and the inflammatory response is exacerbated (71). Moreover, gut microbiota dysbiosis mediates CNS inflammatory responses by damaging the intestinal epithelial barrier (IEB), disrupting intercellular junctions, increasing intestinal permeability, and promoting inflammatory mediator translocation (72). In pre-symptomatic PD patients (e.g., those with RBD), this dysbiosis reduces short-chain fatty acid-producing bacteria, further elevating permeability and facilitating CNS infiltration of inflammatory mediators like LPS and α-Syn (73). Pathological α-Syn aggregation then accelerates DA neuron degeneration and PD progression. Reducing inflammatory responses is therefore crucial to slowing PD advancement.

## 3 The relationship between the VN and the gut-microbiome-brain axis

The VN, as a component of the parasympathetic nervous system, has multiple physiological functions, including regulating immune responses, digestion, heart rate, and emotional control (74). It is also considered a vital connection between the human brain and the gut, and the pathological biomarker associated with PD, α-Syn, may propagate bidirectionally between the gut and brain via the VN (75). The VN may exert therapeutic effects on PD through the MGBA, primarily by regulating brain plasticity, maintaining gut homeostasis, modulating the HPA axis, and exerting anti-neuroinflammatory effects (Table 1).

**Table 1:** Vagus nerve stimulation mechanisms targeting Parkinson’s disease hallmarks via the microbiota-gut-brain axis.

Mechanistic pathway	Observed effects in PD	Key references
1. Brain plasticity modulation	↑ Functional connectivity (parietal–temporal, ACC/mPFC) ↑ TH, VMAT2 expression ↓ Pathological β-oscillations in STN ↑ BDNF/TrkB signaling	Yang et al. (76), Chunlei (77), Zhang et al. (79), Wang et al. (80), Marano et al. (82), Farrand et al. (83), Hosomoto et al. (111), Zhang et al. (127)
2. Gut microbiota and barrier repair	↑ Abundance of SCFA-producers (e.g., Lactobacillus, Bifidobacterium) ↓ Dysbiosis markers ↑ Tight junction proteins (occludin, claudin-5) ↓ Intestinal permeability	Wang et al. (86), Liu et al. (89), Bora et al. (90), Faraji et al. (128)
3. HPA axis reprogramming	↓ Cortisol hyperactivation Modulation of CRF mRNA in PVN of the hypothalamus ↓ CRH/ACTH stress response Restores neuroendocrine homeostasis	Soares et al. (91), van Wamelen et al. (92), Thrivikraman et al. (93), O’Keane et al. (95)
4. Anti-inflammatory effects	↓ TNF-α, IL-1β, IL-6(brain/periphery) ↑ α7nAChR-dependent CAP ↑ Treg / ↓ Th17 cells ↓ Microglial/astrocyte activation	Ghia et al. (96), Kaniusas et al. (97), Farrand et al. (101), Jiang et al. (102), Kin et al. (110), Liu et al. (129)

ACC, Anterior cingulate cortex; mPFC, medial Prefrontal cortex; TH, Tyrosine hydroxylase; VMAT2, Vesicular monoamine transporter 2; STN, subthalamic nucleus; BDNF, brain-derived neurotrophic factor; TrkB, Tropomyosin receptor kinase-B; SCFA, Short-chain fatty acids; CRF, Corticotropin-releasing factor; PVN, Paraventricular nucleus; CRH, Corticotropin-releasing hormone; ACTH, Adrenocorticotropic hormone; TNF-α, Tumor Necrosis Factor-alpha; IL-1β, Interleukin-1 beta; IL-6, Interleukin-6; α7nAChR-dependent CAP, α7 nicotinic acetylcholine receptors-dependent cholinergic anti-inflammatory pathway; Treg, regulatory T cells; Th17 cells, T helper cell 17.

### 3.1 VNS regulates brain plasticity

VNS can optimize brain plasticity, mainly by improving functional connectivity between brain regions, regulating neurotransmitters, and modulating the secretion of neurotrophic factors. A study by Yang et al. found that transcutaneous VNS (tVNS) can protect the integrity of the BBB in ischemic injury rat models and significantly reduce the infarct size caused by ischemic stroke (76). Immediate studies have shown that tVNS can alter the functional connectivity between the parietal and temporal lobes in patients with mild cognitive impairment (MCI) and enhance the activity of multiple brain regions, thereby improving brain function in MCI patients, with the cingulate gyrus possibly being one of the targets regulated by tVNS in MCI (77). Another study showed that transcutaneous auricular VNS (taVNS) can significantly activate the left triangular part of the inferior frontal gyrus (IFG) in PD patients with anxiety, increase the concentration of oxyhemoglobin in this brain region, and thereby improve the patients’ anxiety (78). Dysfunction of the thalamocortical connectivity network is considered the basis of migraine pathophysiology. Clinical research results by Zhang et al. confirmed that taVNS may relieve migraines by increasing connectivity between motor-related thalamic subregions and the anterior cingulate cortex/medial prefrontal cortex, while reducing connectivity between occipital cortex-related thalamic subregions and the postcentral gyrus/precuneus (79). Wang et al. found that right vagus nerve stimulation (RVNS) significantly upregulated the levels of tyrosine hydroxylase (TH) and vesicular monoamine transporter 2 (VMAT2) in the midbrain and reduced α-Syn expression in the SN, exerting neuroprotective effects on brain DA neurons (80). Motor behavior in PD rats was also improved after intervention. Thus, RVNS may be a potential therapeutic option for PD. Some researchers believe that PD-related motor symptoms (such as bradykinesia and rigidity) may be associated with the overexpression of pathological brain rhythms in the β band within the subthalamic nucleus (STN) (81). A study by Marano et al. showed that left taVNS can interact with the right STN circuit, reduce the total β power in the right STN, and effectively improve subclinical gait parameters (Timed Up and Go time, velocity, and variability) in PD patients (82). Other studies have proposed that VNS can exert neuroprotective effects on PD models and improve PD-related behavioral deficits by increasing brain-derived neurotrophic factor (BDNF) and enhancing the survival-promoting mechanisms of its receptor, tropomyosin receptor kinase-B (TrkB) (83).

### 3.2 VNS modulates gut microbiota and repairs the intestinal barrier

The ENS and the VN together constitute the main neural pathways of the MGBA. The ENS, a division of the autonomic nervous system (ANS), communicates with the CNS via sensory and motor neurons and neurotransmitters. This communication is primarily mediated by the VN’s afferent and efferent fibers (84). Previous studies have found that VNS can effectively alleviate intestinal barrier damage and restore intestinal permeability (85). Wang et al. demonstrated that VNS treatment for post-stroke hemiplegia is somewhat effective and can improve BBB and intestinal barrier damage following ischemic stroke (86). VNS can also alter the gut microenvironment and microbiota. Preclinical research has shown that subdiaphragmatic vagotomy (SDV) can lower blood pressure in spontaneously hypertensive rats (SHR) while reducing the abundance of *Defluviitaleaceae* bacteria in feces (87). Another study found that SDV can prevent depressive-like behaviors and gut microbiota dysbiosis in mice treated with LPS, further supporting the role of the VN in the MGBA (88). Liu et al. showed that taVNS can alter the natural course of constipation-predominant irritable bowel syndrome (IBS-C), with 16S rRNA sequencing analysis results indicating that taVNS restored the abundance of *Lactobacillus* and increased the abundance of Bifidobacterium probiotics at the genus level (89). Other studies have shown that electroacupuncture stimulation of the VN can reverse the decrease in *Firmicutes* abundance and increase in *Bacteroidetes* abundance caused by ischemic stroke, thereby improving gut microbiota dysbiosis (86). Additionally, a study on adolescent irritable bowel syndrome showed that subjects with higher abundance of *Blautia* after tVNS had better outcomes. This suggests that patients with specific microbial profiles may be more responsive to tVNS (90). In summary, VNS can influence the ENS to some extent, improve intestinal barrier damage, and maintain the homeostasis of gut microbiota, thereby alleviating brain and gut-related diseases.

### 3.3 VNS modulates the HPA Axis

The VN is a key component of the bidirectional communication between the gut and the brain and is a major regulatory element for sensing the “internal environment” and promoting neuroendocrine responses to maintain gut health. As one of the important pathways for emotional and cognitive regulation, dysregulation of the HPA axis is associated with the induction, exacerbation, or progression of PD (91). Therefore, maintaining normal HPA axis function is of great significance for the prevention and treatment of PD in clinical practice. Previous studies have found that modulating the HPA axis can effectively alleviate PD-related depressive symptoms. van Wamelen et al. proposed that HPA axis hyperactivation can occur in the prodromal stage of PD, manifesting as elevated cortisol levels (92). This is closely related to the occurrence of non-motor symptoms in PD, such as depression, anxiety, sleep, and cognition. They further speculated that modulating cortisol levels might be one of the therapeutic targets for PD-related neuropsychiatric symptoms. VNS is believed to play an important role in maintaining neuroendocrine homeostasis by restoring stress-induced HPA axis responses (93). Keller et al. (94) found that VNS may modulate HPA axis function by increasing corticotropin-releasing factor (CRF) mRNA in the paraventricular nucleus of the hypothalamus (PVN) and the firing of specific PVN CRF neurons, thereby upregulating corticosterone levels. VNS has previously been shown to alleviate chronic depression by modulating the HPA axis and reducing the CRH (corticotropin-releasing hormone)/ACTH (adrenocorticotropic hormone) response (95). VNS can also activate the anti-inflammatory effects of the HPA axis via vagal afferent fibers. In rats treated with vagotomy, inflammatory markers (myeloperoxidase activity, serum amyloid P levels, and interleukin [IL]-1β, IL-6, and TNF-α) were significantly elevated (96). Eugenijus Kaniusas et al. confirmed that taVNS can reduce pro-inflammatory cytokines and regulate lung injury by activating the HPA anti-inflammatory pathway, thereby alleviating acute respiratory distress syndrome caused by Covid-19 (97). In summary, the HPA axis is a key component of the MGBA, and a possible mechanism is that VNS modulates PD-related neuropsychiatric symptoms by regulating HPA axis function via the vagus nerve.

### 3.4 Anti-inflammatory effect of VNS

Recent studies have shown that the VN has anti-inflammatory effects, mediated through multiple pathways, including the cholinergic anti-inflammatory pathway (CAP), the HPA axis anti-inflammatory pathway, and the splenic sympathetic nerve anti-inflammatory pathway (98). The cholinergic system is one of the important pathways through which VNS exerts its anti-inflammatory effects. When external stimuli excite the vagus nerve, the nerve terminals produce the anti-inflammatory parasympathetic neurotransmitter acetylcholine (ACh), which activates the α7 nicotinic acetylcholine receptors (α7nAChR) on monocytes and macrophages. This further activates the intracellular NF-κB signaling pathway and the Janus kinase 2/signal transducers and activators of transcription 3 (JAK2/STAT3) pathway, thereby inhibiting the production of pro-inflammatory cytokines such as IL-1β and TNF-α, ultimately exerting anti-inflammatory effects (99). In recent years, the CAP has been considered one of the therapeutic targets for PD. On the one hand, it has anti-neuroinflammatory properties; on the other hand, it can regulate DA release, prevent the degeneration of DA neurons, and rebalance the direct and indirect signaling pathways in the striatum (100). Previous studies have explored the therapeutic potential of VNS in PD through the CAP pathway in PD rat models. Farrand et al. found that after VNS intervention, PD rats showed significant improvements in motor symptoms and increased expression of TH (101). Additionally, reduced expression of glial fibrillary acidic protein (GFAP) and lonized calcium-binding adapter molecule 1 (Iba-1) in glial cells was observed, indicating decreased inflammatory responses. Similar results were observed in another study, where researchers first injected 6-hydroxydopamine into the medial forebrain bundle of Wistar rats and then performed aVNS treatment. The treatment significantly improved motor deficits in PD rats, increased TH and α7 nAChR expression, and reduced levels of pro-inflammatory cytokines (TNF-α and interleukin-1β [IL-1β]). Moreover, it increased the number of regulatory T cells (Treg) while reducing the number of T helper cell 17 (Th17) (102). These studies suggest that VNS may exert neuroprotective effects against DA neuron damage by inhibiting the inflammatory process and modulating innate immune responses.

The VN is also believed to activate splenic sympathetic nerves via a vagal-sympathetic reflex, releasing NE that binds to splenic lymphocytes, promoting the release of ACh, which in turn inhibits the secretion of pro-inflammatory cytokines from splenic macrophages through a negative feedback loop, thereby exerting anti-inflammatory effects via the splenic sympathetic nerve pathway (103, 104). It has been reported that taVNS may inhibit peripheral inflammatory responses by modulating the α7nAChR/JAK2/STAT3 signaling pathway in the spleen, reducing the release of chemokine C-X-C Motif Chemokine Ligand 1 (CXCL1) and exerting anti-inflammatory effects, thereby improving LPS-induced depressive-like behaviors in rats (105). However, this theory may be controversial. Bratton et al. found in their experiments that the vagal efferent nerves in rats do not synapse with splenic sympathetic neurons nor drive their sustained activity (106). Based on this, Martelli et al. proposed a non-neural connection model between the VN and the spleen (107). They suggested that α7nAChRs are located on the peripheral terminals of splenic sympathetic nerves. When afferent T cells are stimulated by ACh, these terminals release NE, which then acts on β-adrenergic receptors on splenic macrophages, inhibiting their release of TNF-α. Other studies have suggested that VNS may induce the release of ACh in the celiac mesenteric ganglia, which binds to post-synaptic α7nAChRs in the splenic nerve, releasing NE in the spleen to exert anti-inflammatory effects (108).

Moreover, significant degeneration of NE neurons in the LC can also exacerbate the neuroinflammatory process in PD (109). Ittetsu Kin et al. demonstrated in a PD rat model that VNS increased NE in the LC and significantly inhibited the activation of microglia and astrocytes induced by 6-Hydroxydopamine (6-OHDA), exerting a protective effect on DA neurons in the SN (110). This alleviated PD-like motor symptoms and DA neuronal degeneration in rats. Their study found that mild stimulation (0.25–0.5 mA) provided optimal anti-inflammatory and neuroprotective effects in PD rats. Other studies confirmed that continuous afferent vagal stimulation reduces inflammatory neuroglia in the SN, upregulates rate-limiting enzyme density in the LC, and alleviates motor deficits in PD rat models (111).

## 4 Innovative strategies for treating PD with VNS

### 4.1 Stimulation modalities

At the end of the 19th century, American neurologist James Corning first used VNS to treat epilepsy. Although the therapeutic effect was not satisfactory, the concept of VNS was introduced to the world (112). With the continuous development of technology, the clinical efficacy and safety of VNS have been greatly improved. In 1997, the US Food and Drug Administration (FDA) approved the implantable left cervical VNS device for the treatment of refractory epilepsy (TRE) (113, 114). In 2005, VNS was approved for the treatment of treatment-resistant depression (TRD) (115). Cheng et al. proposed that VNS might become a novel therapeutic approach for PD, with neuroprotective effects, and indicated that activation of noradrenergic neurons in the LC may play an important role in VNS treatment for PD (116). The stimulation modalities of the VN have gradually evolved into various forms of non-invasive stimulation. Compared with invasive VNS, non-invasive VNS often has the advantages of high safety, portability, and long-lasting therapeutic effects, although it may be slightly inferior to invasive stimulation in terms of stimulation intensity. Currently, the commonly used tVNS in clinical practice includes taVNS, transcutaneous cervical VNS (tcVNS), and closed-loop taVNS (CL-taVNS). TaVNS is a promising neuromodulatory approach developed based on VNS technology and traditional Chinese medicine auricular acupuncture. This technique can stimulate the cutaneous receptive areas of the auricular branches of the VN to regulate bodily functions and achieve therapeutic effects. In 2010, taVNS was approved in Europe for the treatment of epilepsy and depression, and in 2012, it was approved for the treatment of pain (117, 118). In recent years, taVNS has been considered to have an intervention effect on the occurrence and development of PD. Marano et al. showed that taVNS can effectively improve gait parameters such as stride length, swing amplitude, gait velocity, and gait time in PD patients (119). Other studies have shown that taVNS may exert neuroprotective effects on DA damage in PD models by inhibiting inflammatory evolution and modulating innate immune responses (102). In addition, tcVNS works by stimulating the cervical branches of the VN and is also recognized for the prevention and treatment of epilepsy, primary headache, anxiety, depression, and other diseases (120, 121). Morris et al. conducted a pilot study and found that tcVNS can effectively improve gait abnormalities in PD, especially in terms of gait time and stride length (122). CL-taVNS is an automatically controlled taVNS system, the process of which is regulated by biofeedback signals (such as behavioral changes, respiratory changes, brain activity, etc.) (123). This device can more sensitively adapt to dynamically detectable changes in the clinic, thereby providing personalized taVNS protocols and improving therapeutic efficiency. A recent study showed that electroencephalogram (EEG)-gated taVNS can significantly downregulate delta wave power to treat delirium, indicating that EEG-gated taVNS may be a promising option for intervening in clinical disorders of consciousness (124).

### 4.2 Stimulation parameters

Given the good efficacy and high safety of non-invasive VNS, research on tVNS has gradually increased in recent years. However, there is currently no unified standard for stimulation parameters in clinical practice. Reviewing previous studies on tVNS intervention in PD, we found that in improving PD-related motor disorders, the tVNS stimulation frequency is often set at 20 Hz, the stimulation intensity is usually adjusted according to the sensitivity threshold, and the pulse width is mainly 0.2 ms and 0.5 ms (125). van Midden et al. compared the effects of 25 Hz and 100 Hz taVNS on PD gait and found that a stimulation frequency of 25 Hz was more effective in improving gait disorders, while a frequency of 100 Hz was more significant in increasing arm swing velocity (126). Currently, there are few studies using VNS to treat PD-related non-motor symptoms. In a recent study on taVNS intervention for PD-related anxiety symptoms, an intermittent alternating stimulation pattern of 20 Hz and 4 Hz was used. The results showed that taVNS not only improved anxiety symptoms in PD with anxiety (PD-A) patients but also modulated the function of the IFG (78). In summary, research on different stimulation parameters for improving PD symptoms is relatively lacking. Future studies should explore the optimal stimulation parameters for tVNS targeting different PD symptoms on the basis of proving the efficacy of tVNS, in order to provide more precise and efficient treatment options for PD patients.

### 4.3 Controversies, open questions, and future directions

Despite the promising role of VNS in modulating PD via the MGBA, several controversies and unresolved issues warrant attention. Key controversies include the dual role of GCs, which may promote neuroinflammation in some contexts (e.g., through HPA axis dysregulation exacerbating DA neuronal damage) yet offer neuroprotective effects in others (e.g., by inducing Parkin expression via CREB pathways to prevent neuronal death) (37, 91). Similarly, vagotomy has been proposed as a preventive measure against α-synuclein propagation (16), but it may compromise anti-inflammatory benefits associated with intact vagal function, as vagal ablation can exacerbate inflammatory responses in models like LPS-induced depression (96). Additionally, the mechanisms underlying VNS-mediated anti-inflammatory effects—particularly through splenic sympathetic pathways—remain debated, with conflicting evidence on neural versus non-neural connections (107, 108).

Open questions persist regarding the specificity of VNS effects, such as how it precisely regulates gut microbiota homeostasis and whether these changes directly influence PD progression in humans. The optimal stimulation parameters (e.g., frequency, intensity) for targeting different PD symptoms (motor vs. non-motor) are also underexplored, as current studies show variable efficacy (125, 126). Future research should prioritize personalized VNS therapies, leveraging closed-loop systems integrated with multimodal biomarkers (e.g., EEG-gated taVNS) to dynamically optimize stimulation in real-time (123). Large-scale clinical trials are needed to validate VNS efficacy across diverse PD subtypes and address non-motor symptoms like cognitive impairment and gastrointestinal dysfunction, which have been neglected in prior studies.

## 5 Conclusion

This review synthesizes evidence supporting VNS as a promising intervention for PD through modulation of the MGBA. By targeting key pathways—including gut microbiota homeostasis, HPA axis function, neuroinflammation, and brain plasticity—VNS demonstrates potential to mitigate PD progression across multiple systems.

While preclinical and clinical studies highlight its therapeutic value, translating VNS into clinically actionable strategies requires resolving some controversies. Future efforts should prioritize large-scale trials validating efficacy for both motor and non-motor symptoms, alongside development of personalized closed-loop systems to enhance precision.

In summary, VNS technology holds promise to break through the limitations of traditional PD treatments through mechanism-driven targeted interventions and clinically adaptable technological empowerment, ultimately achieving a paradigm shift from “symptom control” to “disease modification.”
